# A Structured, Auditable Taxonomy for Pharmacist-Identifiable Medication-Related Risk Signals in Routine Admission Data: A Retrospective Medication Safety Audit in a High-Risk Inpatient Cohort

**DOI:** 10.7759/cureus.116074

**Published:** 2026-09-10

**Authors:** Hidenori Sagara, Momoka Yamane, Yasuhide Kurata, Akira Inamori, Hisako Teshima

**Affiliations:** 1 Division of Medical Safety, Faculty of Pharmaceutical Sciences, Sanyo-Onoda City University, Yamaguchi, JPN; 2 Department of Pharmacy, Yamaguchi Prefectural Grand Medical Center, Hofu, JPN; 3 Department of Nursing, Yamaguchi Prefectural Grand Medical Center, Hofu, JPN

**Keywords:** drug-related problems, hospital pharmacy, in-hospital stroke, medication adherence, medication reconciliation, medication safety, transitions of care

## Abstract

Background

Hospital pharmacists collect rich admission data, i.e., medication history, brought-in medicines, prescription and laboratory records, and vital signs, yet the medication-related problems these data contain are surfaced by ad hoc, non-auditable judgment. Existing drug-related problem classifications code a problem after it has been recognized; they do not specify which admission data element to examine or which decision rule to apply. The prespecified aims of this study were to develop a taxonomy of pharmacist-identifiable medication-related risk signals with explicit operational definitions and data sources, and to apply it, as a feasibility application in a very small cohort, to routine admission data from patients who developed in-hospital ischemic stroke. A four-stage pharmacist-led assessment framework was derived post hoc as a secondary output.

Methodology

In this single-center retrospective medication safety audit (Strengthening the Reporting of Observational Studies in Epidemiology), 12 signal categories, each with an operational definition and data source, were fixed before record review and mapped to existing classifications. The taxonomy was applied to 17 consecutive patients with in-hospital ischemic stroke (August 2020-September 2023). Two pharmacists reviewed the records jointly and classified each case by consensus, counting only signals documented before the index date. Analysis was descriptive; category-level counts with explicit denominators were the primary quantities.

Results

All 17 cases could be classified against all 12 categories from admission data. Category-level counts were: poor adherence or medication management, 10 of 17; residual medication discrepancy, 5 of 17; treatment self-discontinuation, 3 of 17; insulin instruction mismatch, 2 of 17; suboptimal blood pressure control, 9 of 13 treated hypertensive patients; and suboptimal glycemic control, 5 of 8 patients with diabetes. As a secondary summary, 14 of 17 patients had at least one signal. Inter-rater reliability, content validity, and pharmacist workload were not measured.

Conclusions

In this 17-patient feasibility application, a predefined, operationally defined taxonomy could be applied to data pharmacists already collect. It yields a potentially feasible structured and auditable framework using routinely available admission data, not a validated or demonstrably low-burden tool. Because no comparator was studied, the counts are neither specific to nor predictive of stroke; independent prospective evaluation of reliability, validity, and workload is required.

## Introduction

Hospital pharmacists are central to inpatient medication safety. At admission, they routinely gather a rich body of information through medication, i.e., history assessment, review of brought-in medicines (patients’ own medications), and medication reconciliation, and have access to prescription records, vital signs, and laboratory data, which they act on through medication review, medication therapy management, adherence support, prescriber consultation, and communication with community pharmacies during transitions of care [1-5]. In Japan, ward-based pharmacist services have been reimbursed since 2012, and admission medication assessment by ward pharmacists is now routine in acute-care hospitals [6]; hence, the data considered here are already collected as part of usual care.

These data contain many medication-related problems relevant to patient safety, including unintended admission discrepancies and suboptimal adherence, which are common in hospitalized patients [7] and remain a priority of the World Health Organization’s patient safety agenda [8]. Yet, pharmacists identify such problems largely through ad hoc clinical judgment that is inconsistent between individuals, is not auditable, and is not comparable across patients or settings. Established classification systems for drug-related problems, namely, the categories of Hepler and Strand [9], the Pharmaceutical Care Network Europe (PCNE) classification [10], and, for reconciliation, the medication discrepancy taxonomy (MedTax) [11], are designed to code a problem, its cause, and the intervention after a clinician has already recognized that a problem exists. They do not specify which admission data element should be examined, or with what decision rule, to decide whether a problem is present. To our knowledge, no classification has been operationalized in that form for the admission data hospital pharmacists routinely collect. This gap limits medication safety auditing, quality improvement, and transparent reporting.

Testing the feasibility of such a method is best done in a well-defined, high-risk inpatient cohort. In-hospital ischemic stroke, i.e., new-onset ischemic stroke occurring after admission for an unrelated condition, affects patients with complex medication regimens and multiple vascular risk factors and is associated with worse outcomes and greater treatment delays than community-onset stroke [12,13], providing a bounded, high-risk cohort already under pharmacist care in which a predefined taxonomy can be applied to routinely collected admission data.

Therefore, this medication safety audit had two prespecified, method-focused aims: first, to develop a taxonomy of pharmacist-identifiable medication-related risk signals with explicit operational definitions and data sources; and second, to apply it, as a feasibility application, to routinely collected admission data from patients who developed in-hospital ischemic stroke, a cohort used here solely as a test bed. A third output, namely, a four-stage pharmacist-led admission medication assessment framework, was not prespecified; it was derived post hoc from the audit and is presented as an illustrative proposal only. We did not aim to determine whether these problems are more common before stroke than in other inpatients, which would require a comparator group, nor to establish the reliability, validity, or clinical utility of the taxonomy, which require separate prospective studies.

## Materials and methods

Study design and setting

This was a single-center, retrospective medication safety audit conducted at a tertiary referral hospital and reported in accordance with the Strengthening the Reporting of Observational Studies in Epidemiology (STROBE) statement [14]. The study period was from August 2020 to September 2023.

Participants and case identification

We included all consecutive patients who developed ischemic stroke during hospitalization in a non-neurological ward and were subsequently transferred to neurosurgery or neurology. Cases were identified from departmental transfer records (transfers from non-neurological wards to neurosurgery or neurology during the study period), and each candidate case was verified against the discharge summary and the imaging report. No institutional stroke registry, diagnostic code (International Classification of Diseases) search, or neurology consultation database was used. Consequently, in-hospital ischemic strokes that were managed on the original ward without transfer, managed by another service, or followed by death before transfer would not have been captured; this ascertainment mechanism is a potential source of selection bias and is discussed in the Limitations. We excluded patients admitted primarily to neurosurgery or neurology and those with stroke symptoms already present at admission.

Definition of in-hospital ischemic stroke and index date

In-hospital ischemic stroke was defined as new-onset ischemic stroke occurring after admission, confirmed by the treating physicians based on clinical findings together with brain imaging (CT and/or MRI); hemorrhagic stroke was excluded by imaging. The index date was defined as the date of the first documented stroke symptom onset.

Data sources

Data sources comprised pharmacist admission interview and brought-in medicine review records, prescription and medication records, vital signs, laboratory data, transfer records, and discharge summaries.

Taxonomy development

Medication-related risk signals were defined as medication use or treatment management issues identifiable from pharmacist-accessible data that could reasonably prompt medication review, adherence support, prescriber consultation, documentation, or communication with community pharmacies. Candidate categories were generated by the two hospital pharmacists who later performed the classification, drawing on the item set of the department’s routine admission medication assessment (medication history interview, brought-in medicine review, and medication reconciliation) and on the problem domains of established classifications [9-11]. Candidate items were reviewed and refined through discussion within the author group (hospital pharmacists, a nurse, and an academic pharmacist in medication safety) and retained only if they satisfied three properties: identifiability from a data source available at admission, an explicit decision rule, and a corresponding pharmacist action. The resulting 12 categories, each with an operational definition and data source (Appendix 1), were fixed in a written classification sheet before record review; categories were not mutually exclusive. Development did not include a systematic literature review, pilot testing on an independent sample, an external expert panel, a formal content validity assessment [15], or public registration; these omissions are acknowledged as limitations and specified as next steps.

A total of 12 categories were specified: poor medication adherence or management; residual medication discrepancy; treatment self-discontinuation; treatment interruption; antithrombotic therapy interruption or discontinuation; antithrombotic non-prescription requiring clinical adjudication; suboptimal blood pressure control; suboptimal glycemic control; insulin dose misunderstanding or instruction mismatch; multiple prescribers or fragmented care; smoking-related or alcohol-related medication management concern; and difficulty despite once-daily dosing or one-dose (blister) packaging. In brief, poor adherence or management was assessed from brought-in-medicine review (residual quantities, packaging) and the admission interview. Suboptimal blood pressure control was defined a priori as an admission blood pressure ≥140/90 mmHg in a patient with treated hypertension, per the 2019 Japanese Society of Hypertension guidelines [16]. Suboptimal glycemic control was defined as a most recent HbA1c ≥7.0% (National Glycohemoglobin Standardization Program) in a patient with diabetes, per the Japan Diabetes Society clinical practice guideline [17]. Antithrombotic status was taken from the documented prescription and any recorded interruption, discontinuation, or non-prescription. The taxonomy was subsequently operationalized post hoc into a structured four-stage pharmacist-led admission medication assessment framework (Figure 1; described in the Discussion).

**Figure 1:**
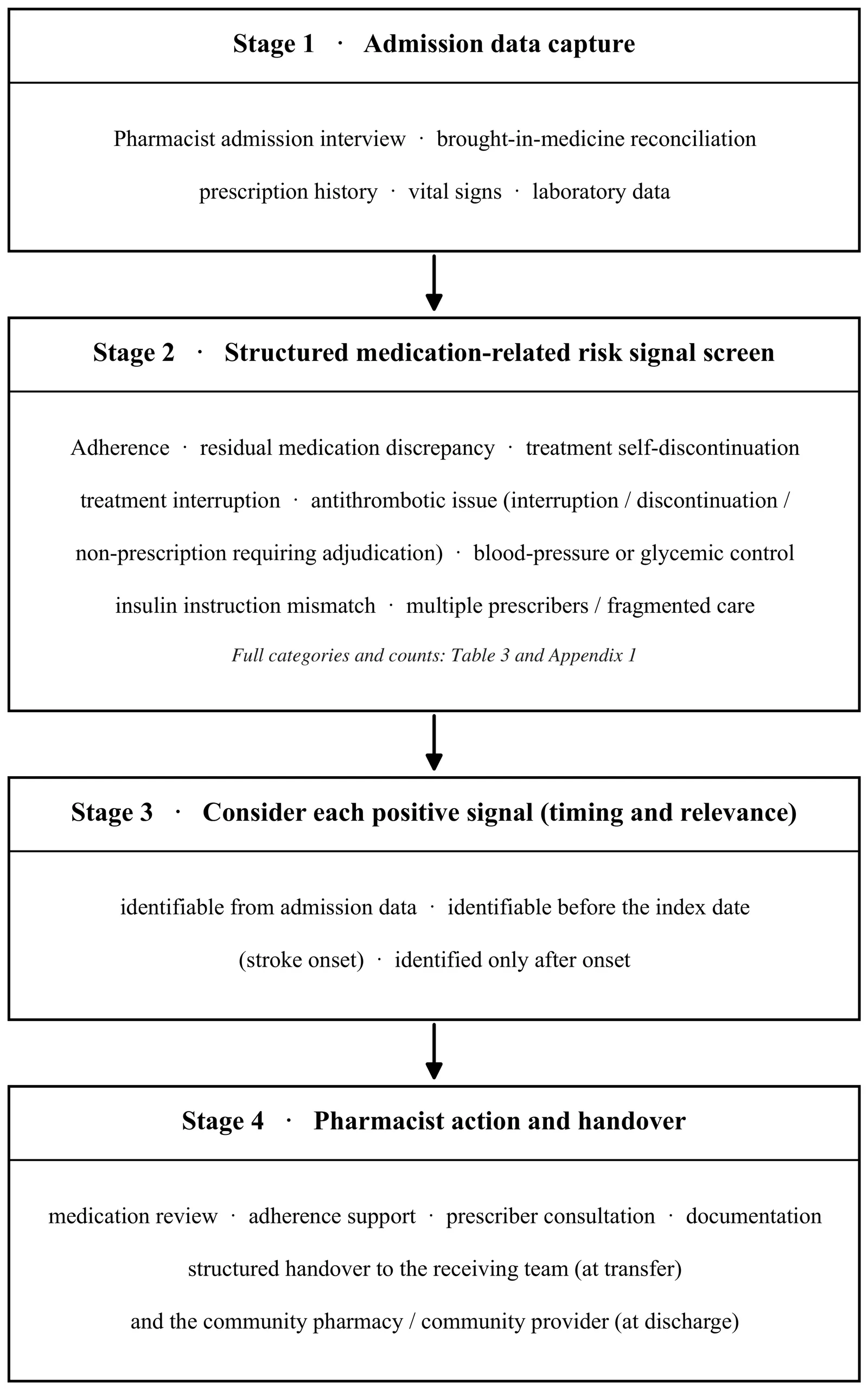
Illustrative candidate framework for structured pharmacist-led admission medication assessment (post hoc proposal). This four-stage framework was assembled post hoc from the audit findings to support identification and management of medication-related risk signals; it was not prespecified. Stage 1 captures pharmacist-accessible admission data. Stage 2 screens for the predefined medication-related risk signal domains. Stage 3 considers each positive signal according to timing and clinical relevance. Stage 4 links positive signals to specific pharmacist actions and to a structured handover to the receiving team and the community pharmacy. The framework is an illustrative proposal; it has not been validated, and its reliability, workload, acceptability, and incremental benefit over usual care have not been measured. Prospective evaluation is required.

Relation to existing classification systems

Each category was mapped to its closest correspondence in the Hepler-Strand categories [9], the PCNE classification V9.1 [10], and MedTax [11]. We assessed whether the category was adapted from an existing system or is, to our knowledge, not separately represented in them. Four points explain why a new, admission data-oriented taxonomy was constructed rather than an existing system adopted unchanged. First, the existing systems are output classifications: they code a drug-related problem, its cause, and the intervention once a clinician has judged that a problem exists, whereas the present taxonomy is an input screen that binds each item to a specific admission data source and an explicit decision rule, so that the basis for each classification can be audited case by case (Appendix 2). Second, seven categories (1, 2, 3, 6, 7, 8, and 9 in Appendix 1) were adapted from existing systems, chiefly the PCNE patient-related and treatment-effectiveness domains, the Hepler-Strand untreated indication category, and MedTax for reconciliation discrepancies, by attaching a data source and threshold. Third, five categories (4, 5, 10, 11, and 12) are not separately represented in those systems: the split of treatment interruption into non-antithrombotic and antithrombotic items, and the contextual items fragmented care, smoking- or alcohol-related concern, and difficulty despite a simplified regimen, were added because each maps to a distinct pharmacist action at admission. Fourth, the taxonomy is intended to be complementary rather than competing: a signal identified by the screen can subsequently be coded in PCNE or MedTax terms for problem, cause, and intervention.

Signal classification and reviewer process

Two pharmacists jointly reviewed the electronic medical records and classified each case against the 12 predefined categories by consensus, using the predefined classification sheet. Per-category counts, each with its relevant denominator, were the primary quantities of interest. A patient was additionally counted as positive for a composite descriptive indicator if at least one signal from any category was identifiable from records dated before the index date (the date of stroke onset). The antithrombotic non-prescription category, which requires clinical adjudication rather than record-based identification, was treated as descriptive only and did not count toward this composite. The composite was an unweighted binary any-signal indicator that combined categories of differing clinical severity, preventability, and denominators; it is reported as a secondary descriptive summary only. To limit hindsight bias, a signal was counted only if supported by a record dated before the index date. Because all patients were stroke cases, the reviewers could not be blinded to the outcome. Outcome-aware interpretation of ambiguous entries therefore cannot be excluded and is addressed as a limitation. Because classification was performed jointly by consensus rather than by independent rating, formal inter-rater reliability (e.g., Cohen’s κ [18]) was not assessed; prospective, independent evaluation of the taxonomy’s reliability is planned.

Vascular risk factors and modified vascular risk burden score

Individual vascular risk factors and a transparent description of background risk are presented in the Results section. In addition, a modified vascular risk burden score was computed solely as a compact descriptive summary: each component contributed 1 point except previous stroke or transient ischemic attack, which contributed 2 (as in CHADS_2_ [19]), giving a possible range of 0-9 (heart disease, hypertension, age ≥75 years, diabetes mellitus, previous stroke or transient ischemic attack (2 points), dyslipidemia, smoking, and alcohol history). This ad hoc score is not a validated prediction model, played no role in signal classification, and is peripheral to the study’s aims.

Statistical analysis

Descriptive statistics were used: continuous variables are summarized as the mean, with median and range where distributions were skewed, and categorical variables as counts. Percentages are reported only where the denominator is the full cohort (n = 17). For categories whose denominator was a subgroup smaller than the full cohort (the antithrombotic, blood pressure, and glycemic control categories, with denominators of 7-13 patients), results are reported as counts with their explicit denominators and without percentages, because a change of a single patient would alter the percentage by 8-14 percentage points and would imply spurious precision. No hypothesis testing was performed, given the small sample and the absence of a control group, and no causal inference was intended.

Ethical considerations

This audit was approved by the institutional clinical research ethics review committee (approval number: 2024-J005) and conducted in accordance with the Declaration of Helsinki and the Japanese Ethical Guidelines for Medical and Biological Research Involving Human Subjects. The requirement for written informed consent was waived, and an opt-out approach was used, with study information disclosed on the institution’s website. All data were anonymized.

## Results

Application of the taxonomy

The predefined taxonomy was applied to all 17 cases; every case could be classified against all 12 categories from the data routinely available at admission, and no classification field was left undetermined. The case-level derivation is provided in Appendix 2.

Patient characteristics and stroke subtype

In total, 17 patients developed in-hospital ischemic stroke (13 men, 4 women; mean age = 79.7 years). All were admitted to non-neurological departments, most commonly cardiology (n = 5). Stroke subtypes were cardioembolic (n = 12), atherothrombotic (n = 3), and lacunar (n = 2). The mean length of stay was 29.6 days (median = 28; range = 7-59). At discharge, six patients returned home, 10 were transferred, and one died (Table 1).

**Table 1:** Patient characteristics and stroke subtype (n = 17). Only the aggregate mean age was available in the audit dataset; individual age values were not available for calculating dispersion. Each cell contains a single data point. All values are descriptive; no hypothesis testing was performed, no statistical tests were applied, and no p-values are reported.

Characteristic	Value
Age, years, mean	79.7
Sex, male, n	13
Sex, female, n	4
Admitting department, cardiology, n	5
Admitting department, orthopedic surgery, n	2
Admitting department, plastic surgery, n	2
Admitting department, general surgery, n	2
Admitting department, gastroenterology, n	2
Admitting department, diabetes and endocrinology, n	2
Admitting department, nephrology, n	1
Admitting department, ophthalmology, n	1
Ischemic stroke subtype, cardioembolic, n	12
Ischemic stroke subtype, atherothrombotic, n	3
Ischemic stroke subtype, lacunar, n	2
Length of stay, days, mean	29.6
Length of stay, days, median	28
Length of stay, days, minimum	7
Length of stay, days, maximum	59
Outcome at discharge, home, n	6
Outcome at discharge, transferred, n	10
Outcome at discharge, died, n	1

Background vascular risk factors

All patients had at least one vascular risk factor. Hypertension and age ≥75 years were each present in 13 of 17 patients, heart disease in 10 of 17, and diabetes mellitus, dyslipidemia, and smoking history in 8 of 17 each (Table 2). The modified vascular risk burden score (mean = 4.8; median = 5; range = 1-8) is shown only as a summary. These characteristics describe background risk and are not the primary finding.

**Table 2:** Vascular risk factors and modified vascular risk burden score (n = 17). Individual risk factors are the primary description of background risk. The modified vascular risk burden score (range 0–9; previous stroke/transient ischemic attack (TIA) weighted 2 points, all other components 1 point) is a compact descriptive summary only, is not a validated prediction model, played no role in signal classification, and is peripheral to the study aims. Construction is detailed in the Materials and Methods section. Each cell contains a single data point. The value denotes the number of patients for the risk factors and the score in points for the modified vascular risk burden score; – indicates not applicable. All values are descriptive; no hypothesis testing was performed, no statistical tests were applied, and no p-values are reported.

Variable	Value	Patients, %
Hypertension, n	13	76.5
Age ≥75 years, n	13	76.5
History of heart disease, n	10	58.8
Diabetes mellitus, n	8	47.1
Dyslipidemia, n	8	47.1
Smoking history, n	8	47.1
Alcohol history, n	7	41.2
Previous stroke or transient ischemic attack, n	7	41.2
Modified vascular risk burden score, mean, points	4.8	–
Modified vascular risk burden score, median, points	5	–
Modified vascular risk burden score, minimum, points	1	–
Modified vascular risk burden score, maximum, points	8	–

Category-level medication-related risk signals identifiable before stroke onset

Counts for all 12 predefined categories, including those not observed, are given in Table 3. Among whole-cohort categories, the most frequent signals were poor medication adherence or management (10 of 17; forgetting to take medicines accounted for 5), residual medication discrepancy (scattered or excess brought-in medicines; 5 of 17), treatment self-discontinuation (3 of 17), and insulin instruction mismatch (2 of 17). Fragmented care across multiple institutions and a smoking-related medication management concern were each recorded once. Non-antithrombotic treatment interruption and alcohol-related concern were not observed, and difficulty despite a simplified regimen was not recorded as a distinct signal because such cases were captured under poor adherence or management. Among the 10 patients receiving antithrombotic therapy before onset, interruption was documented in two and discontinuation in one. Of the seven patients without antithrombotic therapy, adjudication by the two pharmacists against documented indications judged anticoagulation potentially indicated but absent in one patient (valvular disease) and not clearly indicated in the remaining six patients, so non-prescription was considered appropriate and does not imply inappropriate non-use; this review was descriptive and did not contribute to the composite indicator.

**Table 3:** Medication-related risk signals identifiable from pharmacist-accessible data before stroke onset, for all 12 predefined categories (n = 17). Counts are shown for all 12 predefined categories, including those not observed (n = 0), to document the full a priori taxonomy and prevent selective reporting. Patients could have more than one signal. Category-level counts are the primary results; the composite any-signal indicator (at least one signal in 14 of 17 patients; Appendix 2) is a secondary descriptive summary. Percentages are reported only where the denominator is the full cohort (n = 17). For the denominator-specific rows (antithrombotic, blood-pressure, and glycemic control; denominators of 7–13 patients), results are reported as counts with their relevant denominators in the Cases, n and Denominator, n columns, and percentages are omitted because a single patient would alter the value by 8–14 percentage points. Antithrombotic non-prescription was reviewed for appropriateness in the 7 patients not receiving antithrombotic therapy; only 1 (valvular disease) was potentially indicated but absent, and this review did not contribute to the composite indicator. In Appendix 2, the antithrombotic column marks the 3 patients (2 interruptions, 1 discontinuation) for whom an antithrombotic issue was the basis of the composite signal. Operational definitions for every category are provided in Appendix 1. Each cell contains a single data point. Cases, n, and Denominator, n are given in separate columns so that every proportion has an explicit denominator; – indicates that a percentage is not reported because the denominator is a subgroup, or that no further note applies. All values are descriptive; no hypothesis testing was performed, no statistical tests were applied, and no p-values are reported.

Medication-related risk signal	Cases, n	Denominator, n	Cases, %	Note
Poor medication adherence or management	10	17	58.8	–
Forgetting to take medicines	5	17	29.4	Subset of poor medication adherence or management
Residual medication discrepancy	5	17	29.4	Scattered or excess brought-in medicines
Treatment self-discontinuation	3	17	17.6	Clinic attendance and/or medication
Insulin dose misunderstanding or instruction mismatch	2	17	11.8	–
Multiple prescribers or fragmented care	1	17	5.9	Care across two or more medical institutions
Smoking-related medication-management concern	1	17	5.9	–
Alcohol-related medication-management concern	0	17	0.0	Not observed
Treatment interruption, non-antithrombotic	0	17	0.0	Not observed
Difficulty despite once-daily dosing or blister packaging	0	17	0.0	Not observed as a distinct signal
Antithrombotic therapy interruption	2	10	–	Denominator: patients receiving antithrombotic therapy
Antithrombotic therapy discontinuation	1	10	–	Denominator: patients receiving antithrombotic therapy
Antithrombotic non-prescription requiring clinical adjudication	1	7	–	Denominator: patients not receiving antithrombotic therapy
Suboptimal blood-pressure control	9	13	–	Denominator: patients with treated hypertension
Suboptimal glycemic control	5	8	–	Denominator: patients with diabetes mellitus

Blood-pressure and glycemic control

Blood pressure control was suboptimal in 9 of 13 patients with treated hypertension, and glycemic control was suboptimal in 5 of 8 patients with diabetes; given the small denominators, these are reported as descriptive observations without percentages.

Composite any-signal indicator

As a secondary descriptive summary, at least one signal from any category was identifiable before stroke onset in 14 of 17 patients, and three patients had none (Appendix 2). Because this indicator aggregates categories of very different severity and preventability, it should not be read as the principal result; the category-level counts above are the primary findings.

## Discussion

Principal findings

The principal contribution of this audit is methodological. A taxonomy of pharmacist-identifiable medication-related risk signals, fixed before record review with an operational definition and data source for each category, could be applied to all 12 categories in all 17 patients from admission data that pharmacists already collect, and the basis of every classification is documented case by case (Appendix 2). This shows that problems usually surfaced by ad hoc judgment can be recorded in a structured, auditable, and transparently reported way. At the category level, adherence- and reconciliation-related signals, antithrombotic management signals, and risk factor control signals were each identifiable from admission data with their own denominators. The composite any-signal count (14 of 17) is reported for completeness only; because it aggregates categories of very different clinical severity, preventability, and likely relation to outcomes, it is not the evidence on which the method rests.

Position relative to existing classification systems

The taxonomy does not replace established drug-related problem classifications [9,10] or MedTax [11]. Those systems are best suited to coding problems, causes, and interventions after recognition, and they remain the appropriate vocabulary for documenting what was found. The present taxonomy operates one step earlier, specifying which admission data element to examine and which decision rule to apply, so that recognition itself becomes auditable (Table 4). Seven of its categories are adaptations of existing domains with an attached data source and threshold; five are items, i.e., drug class-specific interruption and contextual signals such as fragmented care, that existing systems do not represent separately but that map to distinct pharmacist actions at admission. Sites already using PCNE or MedTax could therefore use the screen as a front end and code its outputs in their existing system.

**Table 4:** Relation of the 12 predefined signal categories to existing drug-related problem classification systems. Category numbers follow Appendix 1. Closest correspondence denotes the domain of each existing system to which the category most nearly relates; the mapping was performed by the authors, is approximate, and does not imply equivalence of definitions. PCNE domains refer to the Pharmaceutical Care Network Europe classification V9.1 (P, problem domains; C, cause domains). MedTax is the medication discrepancy taxonomy for discrepancies identified through medication reconciliation. Origin denotes whether the category was adapted from an existing domain by attaching an admission data source and decision rule (Adapted), or is not separately represented in the systems listed (not separately represented). Each cell contains a single statement; – indicates no close correspondence identified.

Category (Appendix 1 number)	Hepler–Strand category, closest correspondence	PCNE V9.1 domain, closest correspondence	MedTax, closest correspondence	Origin in the present taxonomy
1. Poor medication adherence or management	Failure to receive drug (non-adherence)	C7 Patient-related (drug use by patient)	–	Adapted
2. Residual medication discrepancy	Drug use without indication (duplication; approximate)	C8 Patient transfer related	Discrepancy categories (omission, commission, dose, frequency)	Adapted
3. Treatment self-discontinuation	Failure to receive drug (non-adherence)	C7 Patient-related (patient takes less drug than prescribed or none)	–	Adapted
4. Treatment interruption, non-antithrombotic	–	C4 Treatment duration (approximate)	–	Not separately represented
5. Antithrombotic therapy interruption or discontinuation	–	C4 Treatment duration (approximate)	–	Not separately represented
6. Antithrombotic non-prescription requiring clinical adjudication	Untreated indication	C1 Drug selection (no treatment despite indication)	Omission (approximate)	Adapted
7. Suboptimal blood pressure control	Subtherapeutic dosage (approximate)	P1 Treatment effectiveness (effect of treatment not optimal)	–	Adapted
8. Suboptimal glycemic control	Subtherapeutic dosage (approximate)	P1 Treatment effectiveness (effect of treatment not optimal)	–	Adapted
9. Insulin dose misunderstanding or instruction mismatch	Failure to receive drug (improper use; approximate)	C7 Patient related (patient unable to understand instructions)	–	Adapted
10. Multiple prescribers or fragmented care	–	C8 Patient transfer related (approximate)	–	Not separately represented
11. Smoking-related or alcohol-related medication-management concern	–	–	–	Not separately represented
12. Difficulty despite once-daily dosing or one-dose packaging	–	C7 Patient-related (approximate)	–	Not separately represented

A structured, auditable framework for practice

The taxonomy’s practical output is a structured, four-stage pharmacist-led admission medication assessment framework (Figure 1) that organizes the categories into a workflow. Stage 1 captures pharmacist-accessible admission data; Stage 2 screens for the predefined signal domains and records each as present or absent; Stage 3 considers each positive signal by timing and clinical relevance (for example, prioritizing signals still active at admission, such as an ongoing antithrombotic interruption, over historical signals already resolved); and Stage 4 links positive signals to specific pharmacist actions (for example, a reconciliation discrepancy to prescriber consultation, an adherence signal to adherence support, an insulin instruction mismatch to re-instruction with prescriber notification, suboptimal blood pressure or glycemic control to flagging for the treating team, and an antithrombotic interruption to documented prescriber review) and to a structured handover transmitted at transfer and, at discharge, to the community pharmacy. Because each stage draws on information pharmacists already collect, the framework is expected to add documentation rather than new data collection; this expectation, however, is an assumption and not a result of this study. Pharmacist time per patient, completion rate, workflow disruption, acceptability, usability, false-positive burden, and the proportion of signals that lead to an actionable intervention were not measured. The framework should therefore be regarded as potentially feasible, not as low-burden or readily implementable, until these outcomes have been evaluated prospectively. It was derived post hoc from the audit and is offered as an illustrative proposal; its incremental benefit over usual care is unknown. In practice, Appendix 1 can serve as an admission checklist, with the case-level derivations in Appendix 2 as worked examples, and adopting sites can substitute locally applicable guideline thresholds for the blood pressure and glycemic criteria without altering the taxonomy’s structure. Beyond individual patient care, the framework may also lend itself to departmental quality improvement audits, structured training of early-career pharmacists, and standardized handover documentation, although these uses were not tested here.

What this audit does and does not establish

It is important to separate five properties that are easily conflated. Operational definition was achieved: each category has a written decision rule and data source (Appendix 1). Auditability was achieved: the basis for every classification is recorded at the case level (Appendix 2), so a third party can trace each count to its source. Reliability was not established: because the two pharmacists classified cases jointly by consensus, inter-rater agreement (e.g., Cohen’s κ [18]) could not be computed, and it is unknown whether different pharmacists would classify the same patient consistently. Validity was not established: content validity has not been assessed by an external expert panel using a formal method such as the content validity index [15], and criterion validity against outcomes cannot be tested without a comparator. Clinical utility was not tested: no outcome, workload, or implementation measure was collected. The audit therefore supports a narrow claim that the taxonomy can be applied to routine admission data in a structured and auditable way and no more.

Implications for pharmacy practice

Because pharmacists already collect the information in which these signals appear, structured documentation of them at admission could in principle be embedded in the existing admission workflow so that, when present, these signals consistently trigger closer medication review, medication therapy management, and transitions of care follow-up [1-4]. Framing them as a defined set of pharmacist-checkable items, rather than leaving them to ad hoc judgment, would make the pharmacist’s contribution to medication safety more consistent and auditable. Whether such documentation improves patient outcomes, identifies patients at higher risk, or is acceptable to pharmacists in daily practice was not tested here and would require prospective evaluation.

Medication reconciliation and adherence support

Residual medication discrepancy, treatment self-discontinuation, and poor adherence are core targets of medication reconciliation and adherence support [4,5,20]. In some patients, poor adherence was documented despite simplified regimens (once-daily dosing or blister packaging) and was captured within the poor-adherence category rather than as the distinct simplified-regimen signal, a pattern consistent with prior evidence that regimen simplification does not guarantee adherence and that directly assessing medication-taking behavior adds value; given the few affected patients, this observation is illustrative only [20-22].

Antithrombotic therapy review

Recorded antithrombotic interruption (n = 2) or discontinuation (n = 1) represents a discrete, pharmacist-checkable signal that can prompt documented review and, where appropriate, prescriber consultation. Non-persistence with secondary preventive medication after stroke is common [23], discontinuation of aspirin has been associated with ischemic events [24], and suboptimal adherence and persistence to direct oral anticoagulants remain frequent in atrial fibrillation [25]; our descriptive data cannot speak to any such relationship in these patients. Of the seven patients without antithrombotic therapy, only one had potentially indicated but absent anticoagulation (valvular disease); in the others, non-prescription appeared appropriate. These observations should be interpreted cautiously, and no inference of inappropriate non-use is made.

Continuity of care with community pharmacies

Self-discontinuation, residual discrepancy, and fragmented care often originate before admission; communication with community pharmacies and other providers during transitions of care may support continuity of treatment [2-4].

Interpretation and absence of a comparator

These findings must be interpreted with caution. Medication-related problems such as unintended admission discrepancies and suboptimal adherence are common in general inpatient and chronic disease populations [7,26], and this audit had no stroke-free comparator. The proportion of cases with a signal therefore cannot be interpreted as elevated relative to other inpatients, and the study cannot establish that these signals contribute to in-hospital ischemic stroke. Its contribution is the demonstration that a predefined taxonomy can be applied transparently to data routinely accessible at admission; a comparative, ideally prospective, study would be needed to test whether any signal is more frequent before such strokes than in matched inpatients. The composite indicator is a binary any-signal summary that weights heterogeneous categories equally and ignores signal severity; it should be read as a descriptive floor rather than a rate, and the individual categories (Table 3) should be interpreted in preference to it.

Limitations

This study has several limitations: a single-center design, a very small sample (n = 17), retrospective data collection, the absence of a control group, the lack of an objective adherence scale, possible misclassification or incomplete documentation, the inability to infer causality, limited generalizability, and reliance on a post hoc, externally unvalidated framework. Case ascertainment relied solely on departmental transfer records, verified against discharge summaries and imaging, rather than on a stroke registry or diagnostic code search; in-hospital ischemic strokes managed without transfer, managed by another service, or followed by death before transfer would therefore have been missed, and the 17 cases should not be regarded as a complete census of in-hospital stroke at the institution. The taxonomy was developed by the study team from routine practice and established problem domains without a systematic literature review, pilot testing, an external expert panel, or formal content validity assessment, and it was not publicly registered, so its a priori specification cannot be externally verified. Because all patients were stroke cases, the two reviewers could not be blinded to the outcome, and outcome-aware interpretation of ambiguous entries cannot be excluded. Classification was performed by the two pharmacists jointly, by consensus, rather than by independent rating, so formal inter-rater reliability was not assessed, and retrospective classification still involves clinical judgment, particularly for adherence-related signals and for antithrombotic non-prescription. Single admission blood pressure values may reflect acute illness, pain, anxiety, or the hospital setting rather than chronic control, and the timing of the most recent HbA1c differed among patients; future validation should use standardized measurement windows or longitudinal information for these two categories. The modified vascular risk burden score is non-validated and peripheral to the aims, and the individual risk factors in Table 2 are the more transparent description. Pharmacist time, completion rate, workflow disruption, acceptability, usability, false-positive burden, and the proportion of signals leading to an actionable intervention were not measured, so no claim about burden or implementability can be made. Because this was a single-center, quality improvement-oriented medication safety audit, these constraints are expected for its hypothesis-generating aims, but they preclude causal, comparative, or utility inference. The findings are descriptive and hypothesis-generating only.

## Conclusions

In this feasibility application to 17 patients who developed in-hospital ischemic stroke, a predefined, operationally defined taxonomy of pharmacist-identifiable medication-related risk signals could be applied systematically to data pharmacists already collect at admission, with the basis for every classification documented, and could be positioned against existing drug-related problem classifications. The result is a potentially feasible structured and auditable framework using routinely available admission data, not a validated tool and not one shown to be low-burden. The descriptive counts are neither specific to nor predictive of stroke. Independent prospective evaluation of inter-rater reliability, content validity, pharmacist workload and acceptability, and any incremental benefit over usual care is required before the framework is recommended for practice.

## Appendices

Appendix 1

**Table 5:** A priori operational definitions and data sources for the 12 predefined medication-related risk signal categories. Each cell contains a single definition or a single data-source statement, and no cell is left empty. This appendix contains definitions only; it reports no numerical data, no statistical test, and no p-values.

Signal category	A priori operational definition	Data source
1. Poor medication adherence or management	Documented missed doses, disorganized self-management, or a residual brought-in-medicine quantity inconsistent with the prescribed regimen	Admission interview and brought-in-medicine review
2. Residual medication discrepancy	Scattered, duplicated, or excess brought-in medicines, or a mismatch between brought-in medicines and the documented prescription	Brought-in-medicine review and prescription records
3. Treatment self-discontinuation	Patient-initiated cessation of a prescribed medication and/or of clinic attendance, documented before the index date	Admission interview and prescription/visit records
4. Treatment interruption (non-antithrombotic)	Temporary, clinician- or event-driven hold of a non-antithrombotic medication	Prescription/medication records
5. Antithrombotic therapy interruption or discontinuation	Temporary hold (interruption) or cessation (discontinuation) of a previously prescribed antithrombotic agent, recorded before the index date	Prescription/medication records
6. Antithrombotic non-prescription requiring clinical adjudication	Absence of antithrombotic therapy in a patient in whom it might be indicated; appropriateness adjudicated by the two pharmacists against documented indications (e.g., atrial fibrillation, valvular/mechanical indication)	Prescription records and documented diagnoses
7. Suboptimal blood-pressure control	Admission blood pressure ≥140/90 mmHg (i.e., not meeting target) in a patient with treated hypertension, per the Japanese Society of Hypertension Guidelines for the Management of Hypertension (JSH 2019)	Vital signs (admission)
8. Suboptimal glycemic control	Most recent HbA1c ≥7.0% (NGSP) in a patient with diabetes, per the Japan Diabetes Society clinical practice guideline	Laboratory data
9. Insulin dose misunderstanding or instruction mismatch	Documented discrepancy between the intended insulin regimen and the patient’s understanding or self-administration	Admission interview and medication records
10. Multiple prescribers or fragmented care	Care or prescriptions distributed across two or more medical institutions	Admission interview and prescription records
11. Smoking-related or alcohol-related medication-management concern	Documented smoking or alcohol use judged to materially compromise medication management	Admission interview
12. Difficulty despite once-daily dosing or one-dose (blister) packaging	Poor adherence or management persisting despite a simplified regimen (once-daily dosing or blister packaging)	Brought-in-medicine review and admission interview

Appendix 2

**Table 6:** Case-level derivation of the composite endpoint (medication-related risk signals identifiable before stroke onset). Signal columns: Y = present; N = not present. The any-signal and detectability columns use Yes/No. Each cell contains a single data point and no cell is left empty. All values are descriptive; no hypothesis testing was performed, no statistical test was applied, and no p-values are reported.

Case	Any signal	Poor adherence/management	Residual discrep.	Self-discontinuation	Insulin mismatch	Antithrombotic interruption/discontinuation	Suboptimal blood pressure	Suboptimal glycemia	Fragmented care	Smoking/alcohol	Detectable from admission	Detectable before onset
C1	No	N	N	N	N	N	N	N	N	N	No	No
C2	Yes	Y	Y	N	N	N	Y	Y	Y	N	Yes	Yes
C3	Yes	Y	N	Y	N	N	Y	N	N	N	Yes	Yes
C4	Yes	Y	N	N	Y	N	Y	Y	N	N	Yes	Yes
C5	Yes	N	N	N	N	Y	N	N	N	N	Yes	Yes
C6	Yes	N	N	N	Y	N	N	N	N	N	Yes	Yes
C7	Yes	Y	N	Y	N	N	N	N	N	Y	Yes	Yes
C8	Yes	N	N	N	N	Y	N	N	N	N	No	Yes
C9	Yes	Y	Y	N	N	N	Y	Y	N	N	Yes	Yes
C10	No	N	N	N	N	N	N	N	N	N	No	No
C11	Yes	Y	Y	N	N	N	Y	Y	N	N	Yes	Yes
C12	Yes	N	N	N	N	Y	N	N	N	N	Yes	Yes
C13	Yes	Y	N	N	N	N	Y	N	N	N	Yes	Yes
C14	No	N	N	N	N	N	N	N	N	N	No	No
C15	Yes	Y	N	Y	N	N	Y	N	N	N	Yes	Yes
C16	Yes	Y	Y	N	N	N	Y	Y	N	N	Yes	Yes
C17	Yes	Y	Y	N	N	N	Y	N	N	N	Yes	Yes
